# Risk of Second Primary Thyroid Cancer in Women with Breast Cancer

**DOI:** 10.3390/cancers14040957

**Published:** 2022-02-15

**Authors:** Monika Cieszyńska, Wojciech Kluźniak, Dominika Wokołorczyk, Cezary Cybulski, Tomasz Huzarski, Jacek Gronwald, Michał Falco, Tadeusz Dębniak, Anna Jakubowska, Róża Derkacz, Wojciech Marciniak, Marcin Lener, Karolina Woronko, Dominika Mocarz, Piotr Baszuk, Marta Bryśkiewicz, Steven A. Narod, Jan Lubiński

**Affiliations:** 1 International Hereditary Cancer Center, Department of Genetics and Pathology, Pomeranian Medical University, 71-252 Szczecin, Poland; monika.cieszynska@pum.edu.pl (M.C.); wojciech.kluzniak@pum.edu.pl (W.K.); dominika.wokolorczyk@pum.edu.pl (D.W.); cerazy.cybulski@pum.edu.pl (C.C.); tomasz.huzarski@pum.edu.pl (T.H.); jacek.gronwald@pum.edu.pl (J.G.); tadeusz.debniak@pum.edu.pl (T.D.); anna.jakubowska@pum.edu.pl (A.J.); roza.derkacz@read-gene.com (R.D.); wojciech.marciniak@read-gene.com (W.M.); marcin.lener@pum.edu.pl (M.L.); kw.woronko@wp.pl (K.W.); dm.mocarz@wp.pl (D.M.); piotr.baszuk@pum.edu.pl (P.B.); marta.bryskiewicz@pum.edu.pl (M.B.); 2 Department of Clinical Genetics and Pathology, University of Zielona Góra, 65-417 Zielona Góra, Poland; 3 West Pomeranian Oncology Center, Radiation Oncology Department, 71-730 Szczecin, Poland; mfalco@onkologia.szczecin.pl; 4 Independent Laboratory of Molecular Biology and Genetic Diagnostics, Pomeranian Medical University in Szczecin, 71-252 Szczecin, Poland; 5 Read-Gene S.A., 72-003 Grzepnica, Poland; 6 Women’s College Research Institute, Women’s College Hospital, Toronto, ON M5S 1B2, Canada; steven.narod@wchospital.ca

**Keywords:** thyroid cancer, breast cancer, CHEK2

## Abstract

**Simple Summary:**

The goal of this study was to estimate the risk of thyroid cancer following a diagnosis of breast cancer and to identify (therapeutic and genetic) risk factors for the development of thyroid cancer after breast cancer. We followed 10,832 breast cancer patients for a mean of 14 years for new cases of thyroid cancer. Of the 10,832 women with breast cancer, 53 (0.49%) developed a second case of primary thyroid cancer. The ten-year risk of thyroid cancer was higher in women who carried a CHEK2 mutation (1.5%) than in women who carried no mutation (0.9%). In Poland, following a diagnosis of breast cancer, the risk of thyroid cancer is increased four times, but is increased nine times in women who carry a CHEK2 mutation.

**Abstract:**

The goal of this study was to estimate the risk of thyroid cancer following breast cancer and to identify therapeutic and genetic risk factors for the development of thyroid cancer after breast cancer. We followed 10,832 breast cancer patients for a mean of 14 years for new cases of thyroid cancer. All women were genotyped for three Polish founder mutations in BRCA1 (C61G, 4153delA, 5382insC) and four mutations in CHEK2 (1100delC, IVS2 + 1G/A, del5395, I157T). Information was collected on chemotherapy, radiotherapy, hormonal therapies, and oophorectomy. Of the 10,832 women, 53 (0.49%) developed a second primary thyroid cancer. Based on Polish population statistics, the expected number was 12.4 (SIR = 4.3). The ten-year risk of developing thyroid cancer was higher in women who carried a CHEK2 mutation (1.5%) than in women who carried no mutation (0.9%). The age-adjusted hazard ratio for developing thyroid cancer was 1.89 (0.46–7.79; *p* = 0.38) for those with a CHEK2 protein-truncating mutation and 2.75 (1.29–5.85; *p* = 0.009) for those with a CHEK2 missense mutation.

## 1. Introduction

The number of survivors of breast cancer is increasing worldwide due to wide-scale screening programs and effective therapies [1]. A diagnosis of breast cancer increases the risk of psychosocial distress, cardiovascular disease, and second primary cancers, including those of the contralateral breast, endometrium, ovary, colon, lung, and thyroid [2]. The risk of thyroid cancer increases approximately two-fold in breast cancer survivors [3,4]. Breast cancer is also the most common secondary malignancy among thyroid cancer patients [5].

Women with breast cancer are offered a range of therapies, including radiotherapy, chemotherapy, hormonal therapies (tamoxifen and aromatase inhibitors), and oophorectomy. It is not clear if any of these treatments impact the risk of thyroid cancer. The studies that have looked at radiotherapy conclude that the risk of thyroid cancer is not increased [6,7,8]. It is important to consider the risk of secondary primary cancers in the follow-up of breast cancer survivors and to identify those at the highest risk

The positive association between breast and thyroid cancers suggests a possible common genetic susceptibility in some cases. PTEN mutations predispose women to Cowden Syndrome (which features both thyroid cancer and breast cancers) [9]. CHEK2 is a tumor suppressor gene that participates in DNA repair, cell cycle arrest, and apoptosis in response to DNA damage. CHEK2 mutations are associated with cancers of the thyroid, breast, stomach, and prostate [10,11]. In addition, the missense variant I157T is associated with increased risks of colon cancer and kidney cancer [12,13]. Siolek et al. (2015) studied 468 unselected thyroid cancer patients for the presence of a CHEK2 mutation including 11 with a previous diagnosis of breast cancer [14]. Seven of the eleven patients (63%) patients with both thyroid and breast cancer had a CHEK2 mutation, compared to 16% (73/468) of the thyroid cancer patients without breast cancer (*p* < 0.001). Approximately 6% of breast cancer patients in Poland carry a CHEK2 mutation. We have estimated that a CHEK2 mutation increases the risk of breast cancer by 2.5 times and the risk of thyroid cancer by 3.3 times [14,15]. Women with a BRCA1 mutation are at risk for a range of cancers other than breast cancer, but it has not been established that thyroid cancer is part of the BRCA1 cancer spectrum. It is the purpose of this paper to look at the combination of a history of breast cancer and a cancer-predisposing mutation on the subsequent risk of thyroid cancer.

## 2. Materials and Methods

### 2.1. Study Subjects

We enrolled 10,832 breast cancer patients from throughout Poland in the cohort study. Patients were diagnosed in oncology centers in Bialystok, Bielsko-Biala, Bydgoszcz, Gliwice, Kielce, Koszalin, Krakow, Lublin, Lodz, Olsztyn, Opole, Poznan, Rzeszow, Szczecin, Warszawa, Wroclaw, and Zielona Gora between 1996 and 2014. The clinical information collected included the age of diagnosis, year of breast cancer surgery, lymph node status, tumor size, histopathology, estrogen-receptor status, HER2 status, use of chemotherapy, radiotherapy, hormonal therapy, oophorectomy, and prior history of cancers. In some centers, only women diagnosed under age 50 were recruited (6755 women were diagnosed at or below age 50 and 4114 women with breast cancer diagnosed at or above age 50). Patients with a prior diagnosis of thyroid cancer were excluded from the current analysis (40 patients). All patients signed informed consent to use their blood samples for DNA analysis. The study was presented to the Ethics Committee of the Pomeranian Medical University in Szczecin, Poland, but the project did not require the Ethics Committee’s approval.

### 2.2. Laboratory Analyses

The DNA of peripheral blood lymphocytes of patients was examined for the presence of four founder mutations in the CHEK2 gene, using standard molecular biology techniques. We genotyped three founder mutations in BRCA1 (C61G, 4153delA, 5382insC) using a multiplex-polymerase chain reaction (PCR) assay. These three founder BRCA1 mutations constituted 82% of all identified BRCA1-mutation carriers (303/370 patients) in a large series of 1018 Polish women with hereditary breast cancer who were fully sequenced [16]. The 5382insC mutation in exon 20 and the 4153delA mutation in exon 11 was detected using an allele-specific amplification PCR (ASA-PCR), and the third mutation (C61G), a substitution c.181T > G in exon 5 of BRCA1, was detected using restriction fragment length polymorphism PCR (RFLP-PCR) using Ava II enzyme, using methods described in detail previously.

We also genotyped the four most frequent CHEK2 mutations in the Polish population: Three protein-truncating mutations (1100delC, IVS2 + 1G > A, del5395) and one missense mutation (I157T), which is a missense substitution of an isoleucine for threonine in exon 3 [12]. In a study of 390 Polish men with familial prostate cancer, three founder CHEK2 mutations (1100delC, IVS2 + 1G > A, I157T) constituted 100% (39/39 carriers) of all identified CHEK2- truncating or missense mutations [17]. In the current study, we also genotyped for a founder large deletion of exon 9 and 10 of CHEK2, which is present with a frequency of 0.4% in Poland. We are not aware of the presence of other reccurrent CHEK2 large deletions in Poland. Three mutations in CHEK2 including 1100delC (c.1100delC), IVS2 + 1G > A (c.444 + 1G > A), and I157T (c.470T > C) were genotyped using TaqMan assay (Thermo Fisher Scientific, Waltham, MA, USA) using the LightCycler^®^ Real-Time PCR 480 System (Roche Life Science, Penzberg, Germany). The 1100del C mutation was detected using the forward primer Chk2delC_F GGCAGACTATGTTAATCTTTTTATTTTATGG, the reverse primer Chk2delC_R CAAGAACTTCAGGCGCCAAGT, and two probes: Chk2delC HEX-TTTAGATTACTGATTTTGGGC-BHQ1 and Chk2wt 6-FAM- TTAGATTATGATTTTGGGCAC-BHQ1. The IVS2 + 1G > A mutation was analyzed using the forward primer Chek2_IVS_F ACCGAACATACAGCAAGAAACACTT, the reverse primer Chek2_IVS_R TGACCAAATTACCAGCTCTCCTAGA, and two probes: Chek2_IVS_M HEX-CGGATTTTCAGGATAGGTA-NFQ and Chek2_IVS_wt 6-FAM-TCGGATTTTCAGGGTAGGTA-NFQ. The I157T missense variant was detected using the forward primer Chek2_Mis _F TGTTCTCTATTTTAGGAAGTGGGTCCT, the reverse primer Chek2_Mis _R AAGGTTCCATTGCCACTGTGAT, and two probes Chek2_Mis _M HEX-CTTCTATGTATGCAATGTAAG-BHQ1 and Chek2_Mis _WT 6-FAM-CTTCTATGTATGCAGTGTAAG-BHQ1. The large deletion of exon 9 and 10 of the CHEK2 gene was genotyped using the multiplex-PCR reaction. In detail, two primer pairs were designed specifically for genotyping a large deletion of exons 9 and 10 in a multiplex PCR. The first pair (CHLdel2F TGTAATGAGCTGAGATTGTGC, CHLc2R CAGAAATGAGACAGGAAGTT) flanked the breakpoint site in intron 8. The second pair (CHLdelR GTCTCAAACTTGGCTGCG, CHLcF CTCTGTTGTGTACAAGTGAC) flanked the breakpoint site in intron 10. In mutation-negative cases, only two PCR fragments of 379 and 522 bp were amplified from the wild-type allele. The forward primer of the first pair and the reverse primer of the second pair amplified an additional PCR product of 450 bp in deletion-positive cases.

### 2.3. Statistical Analysis

Patients were followed from the date of diagnosis until the first instance of thyroid cancer, death from any cause, or date of last follow up. The annual risk of thyroid cancer was calculated as the ratio of thyroid cancer events to the total person–years of follow-up. We compared the observed number of thyroid cancers to the expected number of thyroid cancer cases using data from female age-specific thyroid cancer incidence rates in Poland.

The 10- year actuarial risk of thyroid cancer was calculated using the Kaplan–Meier method. In a multivariable model, we estimated the adjusted hazard ratios for the development of thyroid cancer according to the age of diagnosis and treatments received (radiotherapy, chemotherapy, tamoxifen, and oophorectomy). Oophorectomy was modeled as a time-dependent variable. We also included the presence of a CHEK2 mutation and a BRCA1 mutation in the Cox proportional hazards model.

## 3. Results

Among 10,792 breast cancer patients, 53 patients (0.48%) developed thyroid cancer in the follow-up period. The majority of thyroid cancers were papillary carcinomas (45), but there were also three cases of follicular and two cases of medullary carcinoma (for 6% of cases the pathological type was missing). The median time from breast cancer diagnosis to thyroid cancer diagnosis was 6.3 years. The characteristics of the women with and without thyroid cancer are presented in Table 1.

The annual rate of thyroid cancer in the cohort was 54 per 100,000 per year (Table 2) Based on the age-specific annual incidence rates of thyroid cancer in the Polish population, the expected number of thyroid cancers was 12.4 (SIR = 4.27).

Moreover, 914 breast cancer patients (8.3%) carried a CHEK2 mutation (659 missense mutations and 255 protein-truncating mutations (47 1100delC; 91 IVS2 + 1G/A and 117 del5395 mutations) and 502 breast cancer patients (4.6%) carried a BRCA1 mutation.

**Figure 1:**
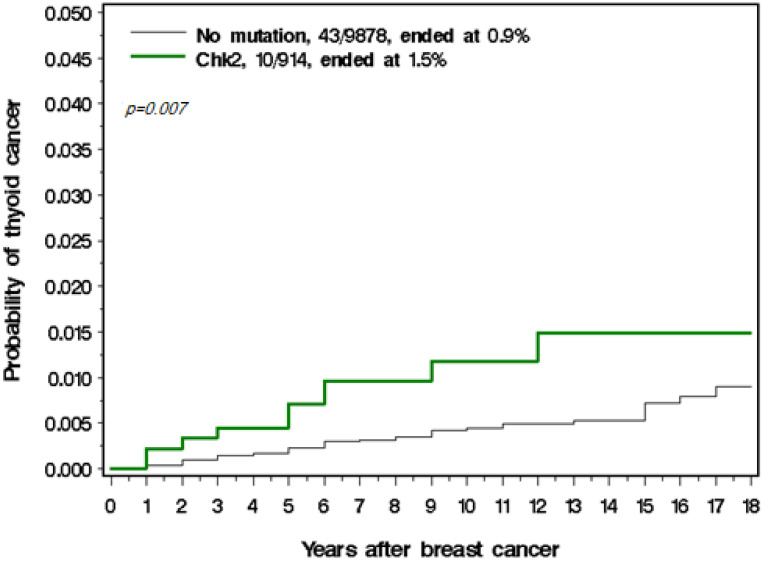
Cumulative incidence of thyroid cancer in those with and without a CHEK2 mutation.

Among the 914 women with a CHEK2 mutation, there were 10 thyroid cancers observed, versus 1.04 expected (SIR = 8.7). Eight of the CHEK2 mutations were missense and two were truncating. The annual risk of thyroid cancer was 114 per 100,000 per year in those with a CHEK2 mutation (Table 3). The cumulative incidence of thyroid cancer in those with and without a CHEK2 mutation is presented in Figure 1. Among the women with a BRCA1 mutation there was only one case of thyroid cancer observed; however, this woman also carried a CHEK2 mutation.

The univariable and multivariable hazard ratios for the development of thyroid cancer are presented in Table 4. The age-adjusted hazard ratio for developing thyroid cancer was 1.89 (0.46–7.79; *p* = 0.38) for those with a CHEK2 protein-truncating mutation and was 2.75 (1.29–5.85; *p* = 0.009) for those with a CHEK2 missense mutation. We did not see an increase in the risk of thyroid cancer associated with radiotherapy. We saw a borderline significant decrease in the risk of thyroid cancer associated with prior chemotherapy (HR = 0.52 (0.28–0.97); *p* = 0.04).

## 4. Discussion

In this large cohort study, we estimated the risk of thyroid cancer in women with a previous diagnosis of breast cancer. There were 53 new cases of thyroid cancer in the cohort for an average annual risk of 0.5% per year. The risk did not vary by age of diagnosis or by attained age. Based on the Polish National Cancer Registry, the expected number of thyroid cancer cases was 12.4 (SIR = 4.3) [18]). Previous studies indicated that breast cancer patients have an approximately two-fold increased risk of thyroid cancer [3; 4]. In Asian populations, the frequency of thyroid cancer after breast cancer is higher (SIR = 4.75) [19,20].

In our cohort, the most significant predictor of thyroid cancer was the presence of a CHEK2 mutation. In our study, 10 of 914 CHEK2 mutation carriers developed thyroid cancer (HR = 2.52; 95% 1.27–5.01; *p* = 0.009). Overall, 10 of the 53 cases of second primary thyroid cancer (19%) could be attributed to carrying a CHEK2 mutation. We have previously estimated that a CHEK2 mutation increases the risk of thyroid cancer by 3.3 times over the general population [14]. Among women with a CHEK2 mutation and breast cancer, the relative risk increases from three-fold to nine-fold.

It is not clear what other factors are responsible for the increase in the risk of thyroid cancer in breast cancer patients, but these might include other genes, treatment factors, and lifestyle factors. We did not test for the presence of mutations in genes other than BRCA1 and CHEK2. It is possible that the genetic background (e.g., SNPs) that predispose CHEK2 carriers to breast cancer also predispose them to thyroid cancer.

We did not see an increase in the risk of thyroid cancer in our cohort associated with radiotherapy, consistent with previous studies [6,7,8]. We saw a borderline decrease in the risk of thyroid cancer associated with previous chemotherapy. The data are few but this observation suggests that chemotherapy does not contribute to the increased risk of thyroid cancer. The association deserves further study. In summary, the increase in thyroid cancer risk does not seem to be due to treatment factors.

It has been suggested that lifestyle factors and environmental factors might contribute to the risk of thyroid cancer among breast cancer patients [21]. Proposed factors include obesity and exposure to endocrine disruptors. In our study, the probability of developing thyroid cancer was similar for those who were ER-positive and those who were ER-negative, and the use of tamoxifen did not reduce the risk of thyroid cancer (Table 4). We did not have information on other risk factors for thyroid cancer such as BMI or lifestyle factors.

There are several other limitations to this study. We assumed that all thyroid cancers were independent cancers (and not breast cancer metastases). We did not have details on the clinical presentation of thyroid cancers nor the treatments they received. For a substantial proportion of women, data on ER and HER2 were missing. We sought founder mutations only, as these account for greater than 80% of the BRCA1 and CHEK2 mutations in the Polish population, but we may have missed a number of mutation carriers [16,17]. It was not practical to perform full gene sequencing on the entire cohort of 10,000 patients.

These results suggest that screening for thyroid cancer among breast cancer patients with a CHEK2 mutation might be warranted. Routine thyroid ultrasonography detects thyroid nodules in 8% of breast cancer patients [22,23]. It is not clear if thyroid cancer screening reduces mortality (which is low), and issues of over-diagnosis have been raised. Further, the risk of thyroid cancers in the CHEK2 carriers was only 1.5% over ten years.

## 5. Conclusions

The risk of thyroid cancer in women with a (truncating or missense) mutation in CHEK2 is approximately nine times that of the general population of Poland. Approximately 19% of thyroid cancers after breast cancer are attributable to CHEK2 mutations.

## Figures and Tables

**Table 1:** Comparison of subjects with or without thyroid cancer after breast cancer.

Variables	No Thyroid Cancer *n* = 10,739	Had Thyroid Cancer *n* = 53	*p*-Value
Year of birth	1955.6 (1918–1993)	1956.6 (1933–78)	0.48
Age at diagnosis Year of diagnosis	51.2 (18–92) 2006.9 (1988–2014)	49.1 (31–75) 2005.7 (1996–2012)	0.17 0.05
Age at thyroid cancer Years from breast cancer to thyroid cancer		55.5 (35–84) 6.3 (1–17)	
ER status positive negative missing	5878 (67.5) 2825 (32.5) 1672	33 (70.2) 14 (29.8) 7	0.70
Her2 status Positive Negative Missing	1239 (17.3) 5929 (82.7) 3571	4 (11.1) 32 (88.9) 17	0.75
Tumor Size <2 cm >2 cm	4251 (50.6) 4143 (49.4)	30 (66.7) 15 (33.3)	0.03
Nodes Positive Negative	3899 (44.6) 4842 (55.4)	10 (21.7) 36 (78.3)	0.002
Chemotherapy yes no	5953 (62.2) 3611 (37.8)	27 (56.3) 21 (43.8)	0.39
Radiotherapy yes no	4574 (59.2) 3158 (40.8)	25 (53.2) 22 (46.8)	0.41
Tamoxifen yes no	6175 (68.1) 2892 (31.9)	37 (77.1) 11 (22.9)	0.18
CHEK2 mutation No Yes	9835 (91.6) 904 (8.4)	43 (81.1) 10 (18.9)	0.006
BRCA1 mutation No Yes	10,220 (95.3) 502 (4.7)	50 (98.0) 1 (2.0)	0.36

**Table 2:** Annual rates of thyroid cancer in the cohort.

	Thyroid Cancer Patients			Poland General Population (Female) Per 100,000 Per Year	
Age Group	Cases Observed	Person Years	Annual Risk Per 100,000 Per Year	Annual Risk (Poland)	Cases Expected
35–40	4	3531	113.2	9.8	0.34
40–44	3	8461	35.4	8.6	0.72
45–49	5	17,812	39.2	12.5	2.22
50–54	15	24,196	70.2	12.5	3.02
55–59	8	20,175	39.6	14.3	2.88
60–64	9	12,389	72.6	17.8	2.20
65–70	6	6067	98.9	16.8	1.02

**Table 3:** Annual rates of thyroid cancer, CHEK2 carriers only.

	Thyroid Cancer Patients			Poland general Population (Female) Per 100,000 Per Year	
Age Group	Number of Events	Person Years	Annual Risk Per 100,000 Per Year	Annual Risk (Poland)	Cases Expected
35–40	1	298	335	9.8	0.03
40–44	0	735	0	8.6	0.06
45–49	1	1538	65.0	12.5	0.19
50–54	2	2067	96.8	12.5	0.26
55–59	2	1692	118	14.3	0.24
60–64	1	1034	96.7	17.8	0.18
65–70	2	489	409	16.8	0.08

**Table 4:** Hazard ratios for thyroid cancer after breast cancer, selected variables.

Variables	Control/Case	Univariate HR(95%CI)P	Multivariate HR(95%CI)P
Age at breast cancer diagnosis <45 45–50 50–65 >65	3419/17 3253/22 2687/11 1308/3	1 1.32 (0.70–2.49) 0.38 1.31 (0.60–2.88) 0.50 0.77 (0.22–2.68) 0.68	1 1.24 (0.66–2.35) 0.51 1.00 (0.44–2.27) 1.00 0.50 (0.14–1.81) 0.29
ER-negative ER-positive	2825/14 5878/33	1 1.18(0.63–2.20) 0.61	
Her2 negative Her2 positive	5929/32 1239/4	1 0.58(0.18–1.92) 0.37	
BRCA1 No Yes	10,220/50 502/1	1 0.38 (0.05–2.72) 0.33	1 0.39 (0.05–2.99) 0.37
CHEK2 No Yes Yes, truncating Yes, missense	9835/43 904/10 242/2 662/8	1 2.52 (1.27–5.01) 0.009 1.89 (0.46–7.79) 0.38 2.75 (1.29–5.85) 0.009	1 2.17 (1.08–4.37) 0.03 1.68 (0.40–7.04) 0.48 2.24 (1.09–5.01) 0.03
Chemotherapy No Yes	3611/21 5953/27	1 0.70 (0.40–1.25) 0.23	1 0.52 (0.28–0.97) 0.04
Radiotherapy No Yes	3158/22 4572/25	1 0.91 (0.50–1.62) 0.76	1 0.96 (0.53–1.72) 0.88
Tamoxifen No Yes	2897/11 6175/37	1 1.60 (0.82–3.15) 0.17	1 1.40 (0.70–2.79) 0.34
Oophorectomy No Yes	7539/37 1162/10	1 1.40 (0.48–1.65) 0.71	1 1.33 (0.61–2.93) 0.48

Oophorectomy was used as a time-dependent variable; in the univariate analyses, missing data on oophorectomy were excluded and in the multivariate analyses the missing data were included in the no-oophorectomy group so that all 10,792 subjects were used in the multivariate regression. Because of collinearity, we did not include ER status or HERs status in the multivariate model.

## Data Availability

The datasets used and/or analyzed during the current study are available from the corresponding author on reasonable request.
